# Isoproterenol Induced Insulin Resistance Leading to Diabetic Ketoacidosis in Type 1 Diabetes Mellitus

**DOI:** 10.1155/2018/4328954

**Published:** 2018-12-17

**Authors:** Ryan Hoff, Chung-Kay Koh

**Affiliations:** Advocate Lutheran General Hospital, Park Ridge, IL, USA

## Abstract

Isoproterenol is known to cause insulin resistance and is often used to treat bradyarrhythmias from atrioventricular block. We report a case of isoproterenol induced diabetic ketoacidosis in a 77-year-old female patient treated with isoproterenol for atrioventricular block prior to insertion of permanent pacemaker. Diabetic ketoacidosis (DKA) developed within hours of starting an isoproterenol drip, and there were no other precipitating factors at that time. DKA resolved quickly after discontinuing isoproterenol and starting insulin drip. DKA is a common complication of diabetes mellitus, with about 140,000 hospital admissions for DKA in 2009. While the rate of DKA has increased by nearly 50% between 1988 and 2009, the rate of mortality has decreased. There are many causes of diabetic ketoacidosis, such as medication noncompliance, infection, pancreatitis, stroke, myocardial infarction, and many others. Isoproterenol may lead to diabetic ketoacidosis by increasing insulin resistance.

## 1. Introduction

Diabetic ketoacidosis (DKA) is a potentially life-threatening complication of diabetes mellitus. There are numerous known precipitants of DKA, including infection, stroke, myocardial infarction, insulin noncompliance, and medications. Diabetic ketoacidosis occurs at a higher rate amongst younger patients, females, those with lower socioeconomic status, those with poor glycemic control, and those with psychiatric symptoms or depression [1]. Presenting symptoms often include nausea, vomiting, polyuria, and polydipsia. Abdominal pain may occur in 46 percent of patients with DKA [2]. Physical exam may reveal Kussmaul respirations, signs of volume depletion, and findings associated with the precipitating cause of DKA. In some cases, a fruity odor is noted. We describe a case of DKA in a woman with type 1 diabetes mellitus, which was caused by medication-induced insulin resistance.

## 2. Case Report

**Figure 1:**
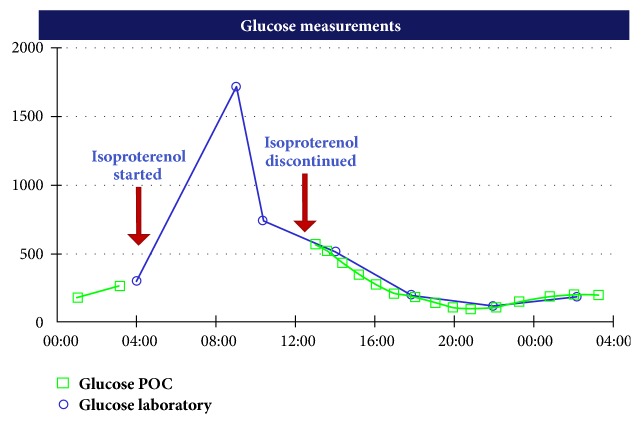
Graph of glucose curve. The blue curve indicates the laboratory glucose measurements (x axis), while the green curve represents the point of care glucose (POC). During infusion of isoproterenol, point of care glucose measured >600 mg/dL.

A 77-year-old woman with a history of type 1 diabetes presented to the endocrinology clinic complaining of lightheadedness for several weeks. That morning, she experienced syncope and fell to the ground, striking her head. There were no episodes of severe or symptomatic hypoglycemia at home. She was directed to the emergency room, where a head CT revealed no evidence of hemorrhage and an electrocardiogram showed sinus tachycardia. Blood glucose was 34 mg/dL, so she was treated with intravenous dextrose, 25 grams. The patient was admitted to the telemetry floor. Over the subsequent 12 hours, blood glucose was monitored closely and remained between 179 and 303 mg/dL. She was treated with insulin glargine 24 units and insulin lispro 4 units TID with meals. Additional medications included enoxaparin prophylaxis, ezetimibe, fluoxetine, levothyroxine, lisinopril, potassium chloride, and pravastatin. With elevated blood glucose, she was treated with an additional dose of insulin lispro 5 units. The telemetry monitor demonstrated several 6- to 9-second episodes of asystole, with intact P waves. She was transferred to the medical intensive care unit for atrioventricular block and started on an isoproterenol drip. Initial laboratory studies were notable for glucose of 297 mg/dL but otherwise normal. Four hours later, bedside blood glucose measured glucose >600 mg/dL (see Figure 1). Repeat laboratory data showed sodium 99 mmol/L, bicarbonate 11 mmol/L, anion gap 20, and glucose 1,713 mg/dL. Glycohemoglobin was 7.5%. Thyroid stimulating hormone was normal. The patient was started on an insulin drip for diabetic ketoacidosis. The isoproterenol was discontinued, and a pacemaker was placed. One hour after the discontinuation of isoproterenol, laboratory studies showed sodium 138 mmol/L, potassium 3.9 mmol/L, serum bicarbonate 17 mmol/L, chloride 103 mmol/L, and glucose 510 mg/dL. Venous blood gas revealed pH 7.19, pCO2 44 mmHg, and bicarb 16 mmol/L. Five hours after the discontinuation of isoproterenol, the diabetic ketoacidosis resolved.

## 3. Discussion

Isoproterenol is a nonselective beta-adrenoceptor agonist and an effective medication for bradyarrhythmias. We used this agent temporarily to increase the patient's heart rate, pending the placement of a permanent pacemaker. As a beta agonist, insulin resistance is a possible effect of isoproterenol. Thus, we monitored the patient's blood glucose closely, which we observed to increase dramatically, rapidly leading to diabetic ketoacidosis. Fortunately, this metabolic derangement was quickly controlled with continuous insulin infusion and discontinuation of isoproterenol. The following day, she was transitioned to subcutaneous insulin, with good control of blood glucose.

Isoproterenol is known to cause glycogenolysis and insulin resistance. It increases serum glucose levels by promoting glycogenolysis, which occurs in a cyclic-GMP mediated fashion. Insulin resistance may be induced by isoproterenol via several mechanisms, including inhibition of resistin gene expression [3], inhibition of insulin mediated tyrosine phosphorylation of the insulin receptor [4], inhibition of localization of the glut-4 receptor to the cell wall [5], and activation of gene expression of SOCS-3 (suppressor of cytokine signaling-3), a negative regulator of insulin [6]. In addition, isoproterenol is known to have counterregulatory effects on sodium potassium chloride channels in skeletal muscle [7].

Tumor necrosis factor (TNF) alpha is produced by adipocytes and is known to cause lipolysis and insulin resistance. Elevated levels of TNF are observed with obesity. Isoproterenol causes a significant increase in TNF alpha levels [8] which may contribute to the medications' impact on overall insulin resistance.

By increasing insulin resistance, isoproterenol may increase the risk of diabetic ketoacidosis. Early recognition and prompt treatment of diabetic ketoacidosis are crucial, because the condition may lead to cerebral edema, severe hypokalemia, and death. Knowledge of this possible adverse event may assist clinicians in the prompt recognition of the complication and may improve outcomes and shorten hospital stays.

Diabetic ketoacidosis is characterized by hyperglycemia, ketonemia, and anion gap metabolic acidosis. While mortality has dramatically improved since the discovery of insulin, all-cause mortality in patients with DKA may be as high as 14%, and the condition is associated with significant morbidity. Several medications have been implicated in precipitating diabetic ketoacidosis, including clozapine, olanzapine, cocaine, lithium, corticosteroids, sodium-glucose cotransporter-2 (SGLT2) inhibitors, and terbutaline. To our knowledge, this case represents the first report of isoproterenol causing diabetic ketoacidosis, which likely occurred via insulin resistance.
